# Supplementary material for: Shear stress-exposed pulmonary artery endothelial cells fail to upregulate HSP70 in chronic thromboembolic pulmonary hypertension
Source: PLoS One. 2020 Dec 3;15(12):e0242960. doi: 10.1371/journal.pone.0242960 (PMC7714249; doi:10.1371/journal.pone.0242960)
Supplement: S1 File — (PDF) [file pone.0242960.s001.pdf]

Figure 2.A.

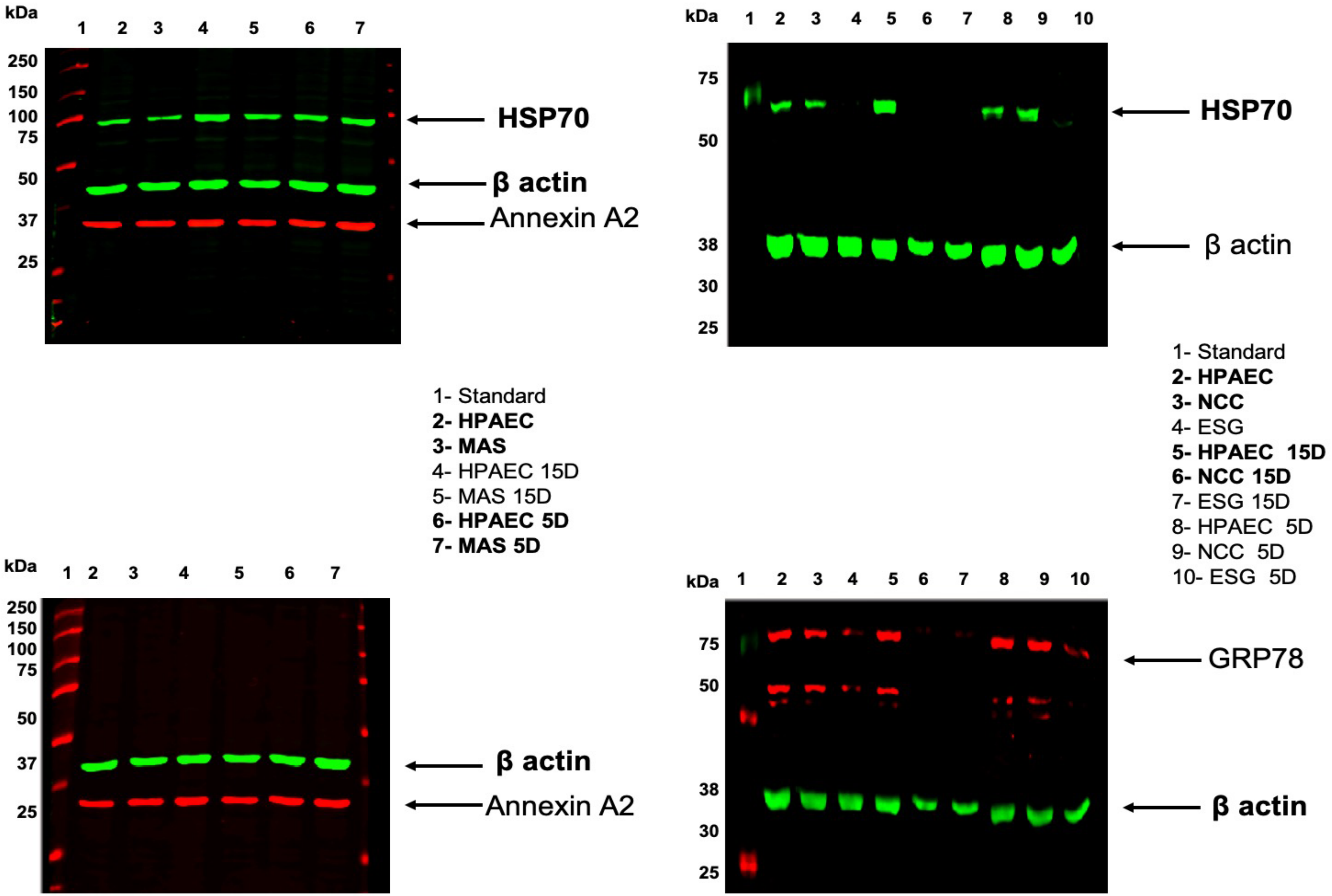

CTEPH-EC list

MAS – CTEPH-EC 1 (patient one)
NCC – CTEPH-EC 2 (patient two)
ESG – CTEPH-EC 3 (patient three)
ASM – CTEPH-EC 4 (patient four)
FSS – CTEPH-EC 5 (patient five)
JEC – CTEPH-EC 6 (patient six)
OJC – CTEPH-EC 7 (patient seven)

Figure 2.B.

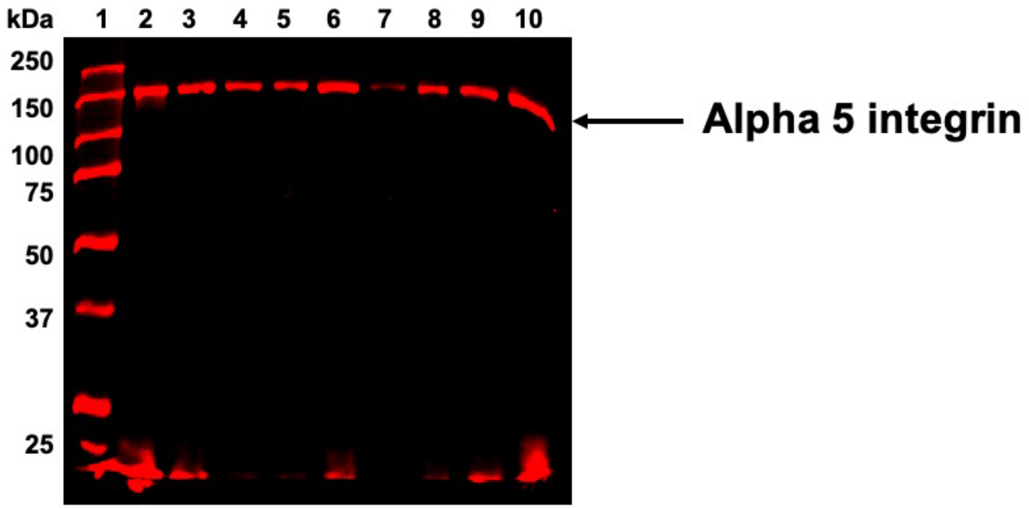

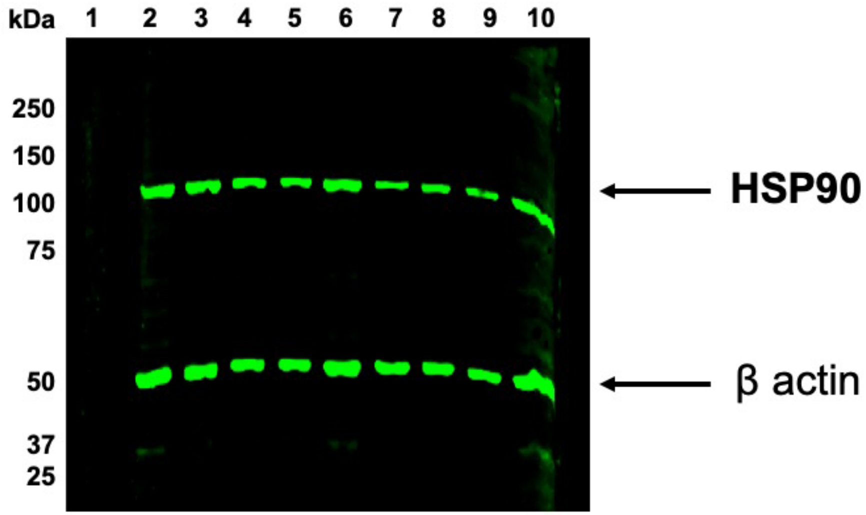

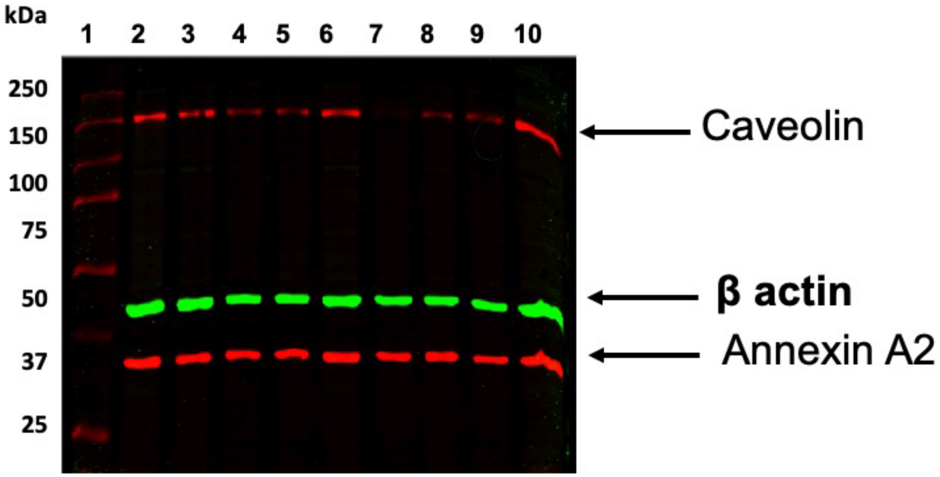

1- Standard
2- HPAEC
3- HPAEC
4- HPAEC
5- ASM
6- NCC
7- ESG
8- FSS
9- JEC
10- JEC

CTEPH-EC list

MAS – CTEPH-EC 1 (patient one)
NCC – CTEPH-EC 2 (patient two)
ESG – CTEPH-EC 3 (patient three)
ASM – CTEPH-EC 4 (patient four)
FSS – CTEPH-EC 5 (patient five)
JEC – CTEPH-EC 6 (patient six)
OJC – CTEPH-EC 7 (patient seven)

Figure 3.A.

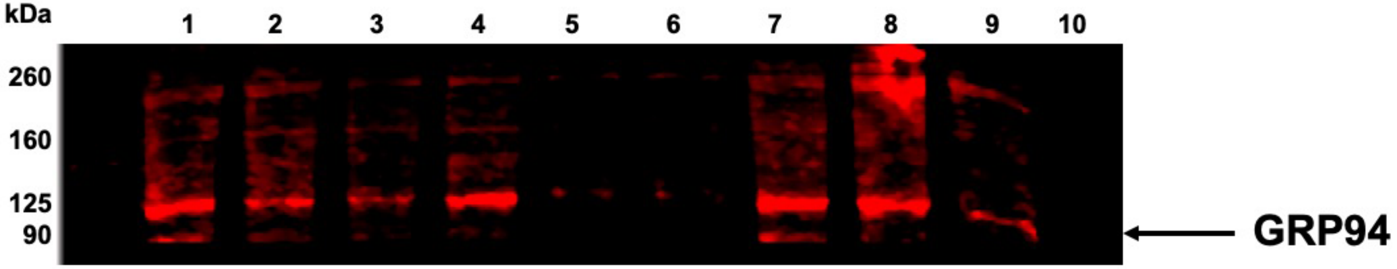

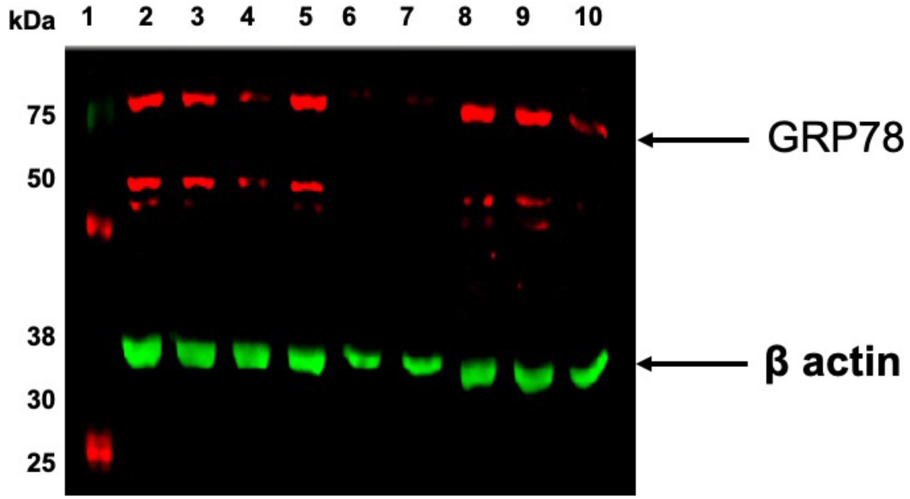

1- Standard
2- HPAEC
3- NCC
4- ESG
5- HPAEC 15D
6- NCC 15D
7- ESG 15D
8- HPAEC 5D
9- NCC 5D
10- ESG 5D

CTEPH-EC list

MAS – CTEPH-EC 1 (patient one)
NCC – CTEPH-EC 2 (patient two)
ESG – CTEPH-EC 3 (patient three)
ASM – CTEPH-EC 4 (patient four)
FSS – CTEPH-EC 5 (patient five)
JEC – CTEPH-EC 6 (patient six)
OJC – CTEPH-EC 7 (patient seven)

Figure 3.B.

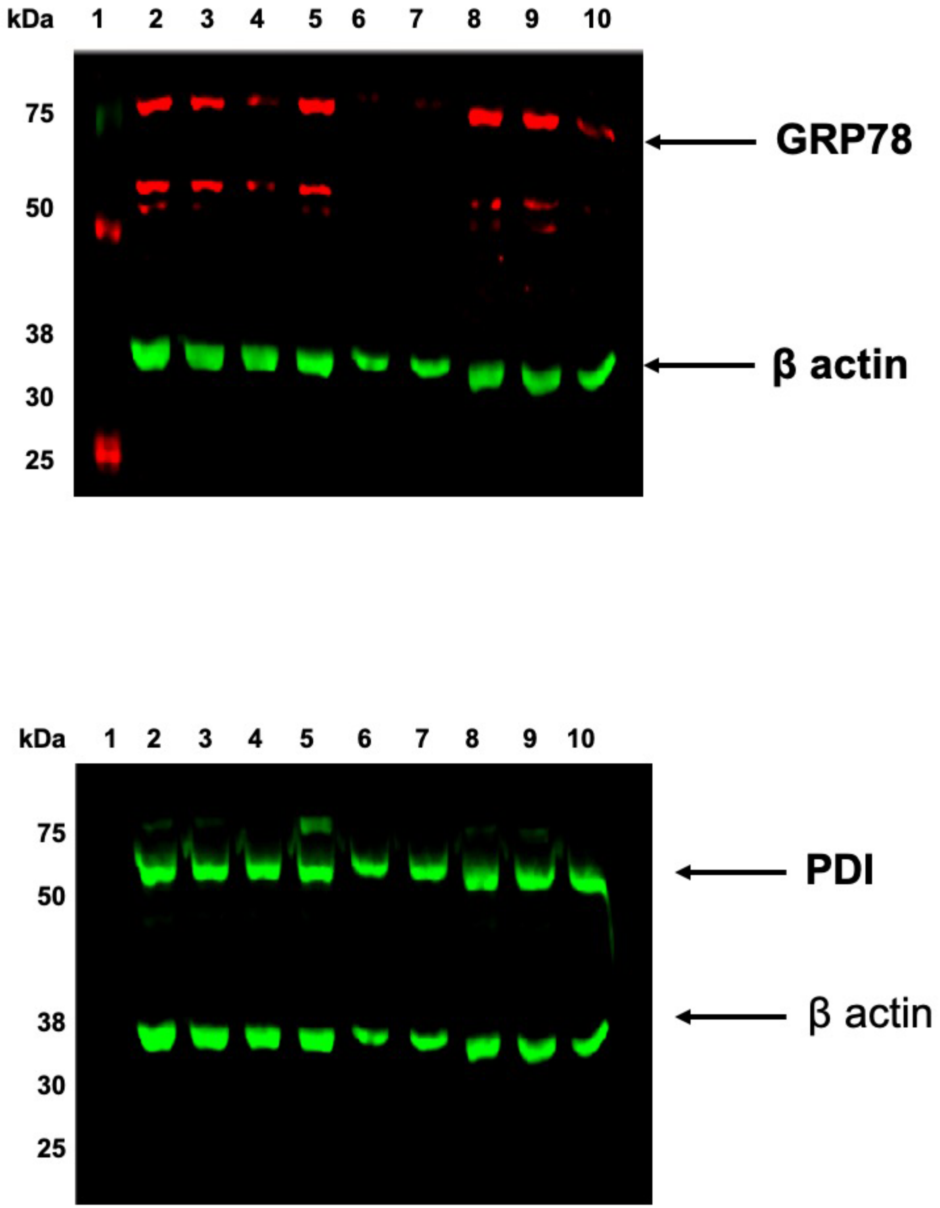

1- Standard
2- HPAEC
3- NCC
4- ESG
5- HPAEC 15D
6- NCC 15D
7- ESG 15D
8- HPAEC 5D
9- NCC 5D
10- ESG 5D

CTEPH-EC list

MAS – CTEPH-EC 1 (patient one)
NCC – CTEPH-EC 2 (patient two)
ESG – CTEPH-EC 3 (patient three)
ASM – CTEPH-EC 4 (patient four)
FSS – CTEPH-EC 5 (patient five)
JEC – CTEPH-EC 6 (patient six)
OJC – CTEPH-EC 7 (patient seven)
