# Supplementary material for: Shear stress-exposed pulmonary artery endothelial cells fail to upregulate HSP70 in chronic thromboembolic pulmonary hypertension
Source: PLoS One. 2020 Dec 3;15(12):e0242960. doi: 10.1371/journal.pone.0242960 (PMC7714249; doi:10.1371/journal.pone.0242960)
Supplement: Attachment — Submitted filename: Salibe et al response to reviewers.pdf [file pone.0242960.s002.pdf]

# Response to reviewers

**Reviewer #1:** The authors showed a paradoxical reduction in the level of Hsp70 protein in shear-stress exposed pulmonary artery endothelial cells from chronic thromboembolic pulmonary hypertension. While their findings are interesting and intriguing, however, I have some concerns:

We are truly grateful to this reviewer for constructive analyses of our work and suggestions.

1) The authors claimed that the decreased hsp70 level was due to shear-stress induced proteostatic dysregulation. Though hsp70 is a major player in the protein quality control system, other proteins including CHIP, an E3 ubiquitin ligase play crucial roles. The authors cannot justify decreased hsp70 to increased protein degradation. If the authors want use the word proteostasis, they will have to back it up with data that decreased hsp70 expression is due to transcriptional or post transcriptional mechanism, both of which can be due to enhanced hsp70 degradation by ubiquitin-proteasomal signaling pathways.

We are grateful for this point of view, and we would like to clarify some points. First, we observed a clear decrease not only in the HSP70 expression in endothelial cells from chronic thromboembolic pulmonary hypertension patients (CTEPH-EC) but also in GRP78, ER HSP70 family member, cytosolic HSP90 and its ER paralog (GRP94). Although we did not investigate if these results are due transcriptional or post-transcriptional levels or either if CHIP is involved, we showed that levels of PDIA1 and alpha 5 integrin were unaltered, suggesting that the downregulation of HSP expression is specific to some extent.
Of note, we have a limited quantity of CTEPH-EC due to their low turnover and bigger size vs normal human pulmonary artery endothelial cells (HPAEC). Then, we have decided focus in performed experiments and tried to understand physiological endothelial cell outcomes in CTEPH-EC and HSP70 function in these related processes as reviewers suggested below. We have now modified the manuscript and removed most of the discussion about proteostasis.

2) The authors assessed the levels of Hsp70 in HPAECs and HPAEC from patients with chronic thromboembolic PH. However they failed to mention the specific isoform of hsp70. So far, there are about 12 -15 hsp70 isoforms, of which most of them are localized to the cytosol.

We apologize for this error, which it has now been corrected, as shown above. The antibody ab5439 used recognizes both constitutively expressed cytoplasmic HSC70 and stress-inducible (HSP70), both referred in the manuscript as HSP70. The text reads as follows:

**Pag 10 / Line 240:** Interestingly, CTEPH-EC had diminished expression of constitutively expressed cytoplasmic HSC70 (HSPA8) and stress inducible form HSP70 (HSPA1A/B) both collectively refereed here as HSP70 under static condition (Fig. 2A).

3) Importantly, what is the relevance of decreased hsp70 in the context of the pathogenesis of chronic thromboembolic PH?

We are grateful for this question, which helped us looking for EC outcomes relevant to the pathogenesis of CTEPH-EC. It is important to highlight that there is scarce information about CTEPH pathophysiology. Despite the thrombus being removed by endarterectomy (PEA), residual HP remains in some patients (30%) bring endothelial dysfunction and smooth muscle cell changes contribute to CTEPH. We have now provided two new experiments to evaluate the endothelial function and HSP70 role through specific HSP70 pharmacological inhibition. The results could be seen below.

**Page 12 / Line 293:** Endothelial dysfunction was proposed to be an essential component in CTEPH (4), however it has not been directly investigated in CTEPH patients. We took advantage of our successful isolation of endothelial cells to evaluate its physiological responses. Interestingly, CTEPH-EC presented strong impairment in migration (Fig. 5) and tube formation (Fig. 6), with less total branches and more cells in each node consistent with frustrated angiogenic response. Moreover, both responses were significantly decreased by the specific (27) HSP70 inhibitor VER155008 (Fig. 5 and 6), clearly showing that HSP70

plays an essential function in migration and angiogenesis both in HPAEC and CTEPH-EC.

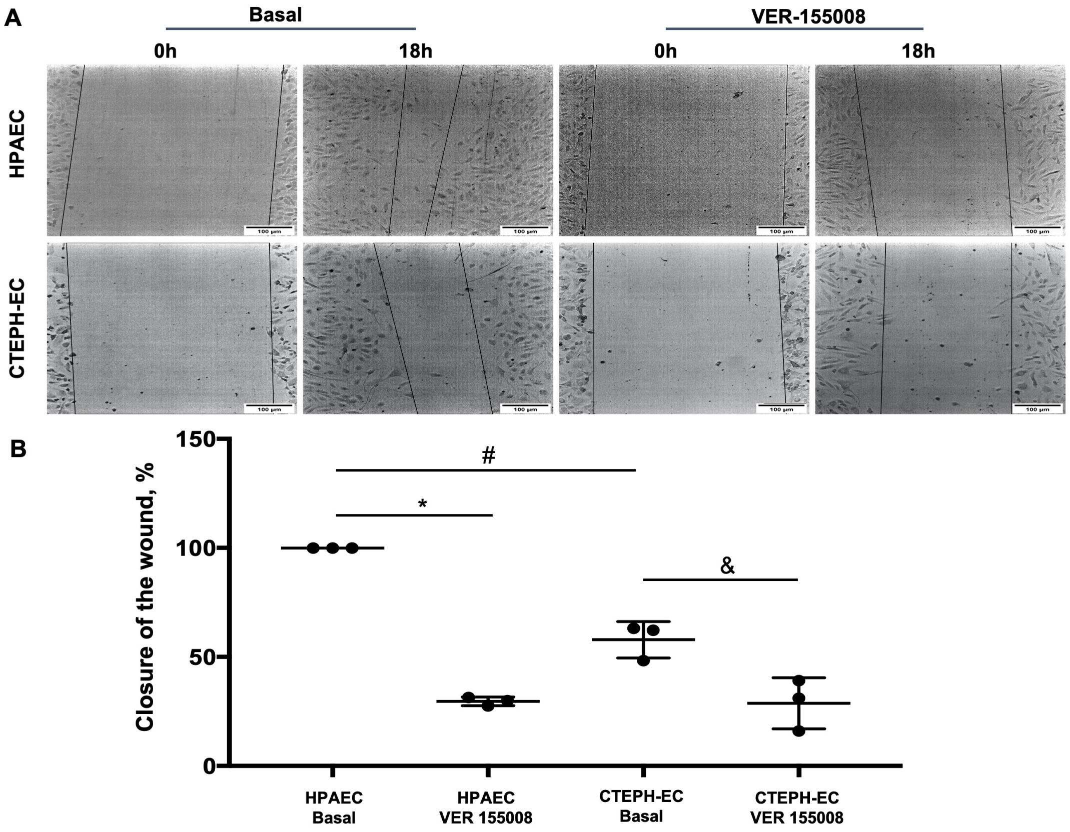


**Fig 5. Migration capacity of HPAEC and CTEPH-EC in basal and with HSP70 inhibitor VER-155008.**

(A) Representative images of HPAECs and CTEPH-ECs basal and after VER-155008. (B) Quantification of the migration. Data are presented as the mean ± standard error: (*) HPAEC Basal vs HPAEC VER, (#) HPAEC Basal vs CTEPH-EC Basal, (&) CTEPH-EC Basal vs CTEPH-EC VER, $P < 0.05$. (n=3).

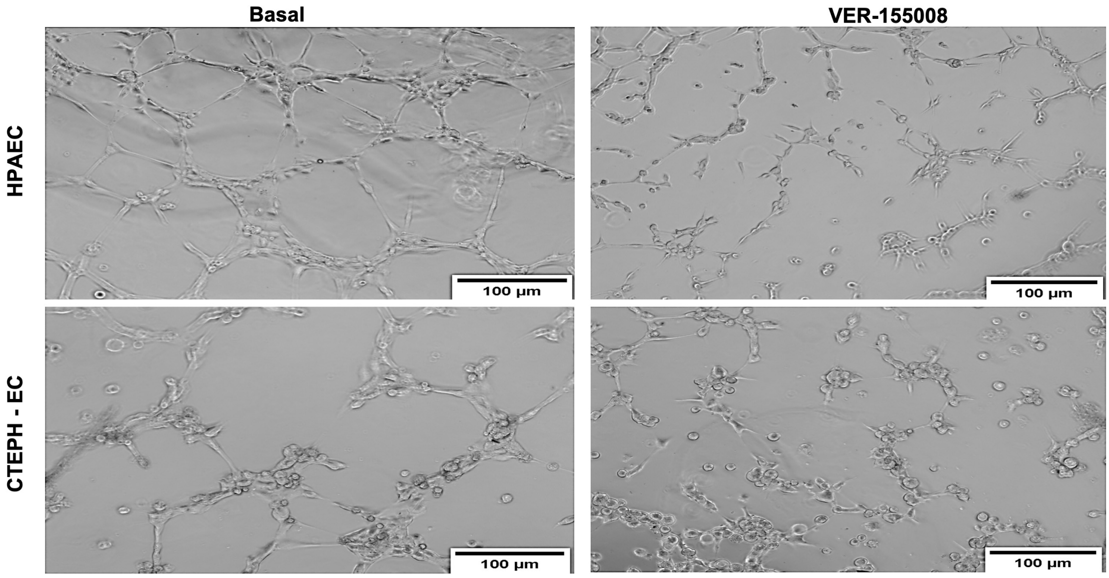


**Fig 6. Tube formation:** representative light microscopic images of the structures formed by HPAEC and CTEPH-EC seeded and incubated for 5h30 min in the absence or presence of 50 uM VER-155008 (n=6).

4) The manuscript was written poorly with no flow to the story. It needs to be re-written and thoroughly edited for clarity.

We fully agree that the manuscript required extensive review. We have now essentially rewritten the whole manuscript and hope it reads now better.

**Reviewer #2**: this study from Saline-Filho et al evaluates the effects of shear stress on EC isolated from CTEPH patients compared to normal control PAEC. The role of proteostasis in pulmonary disease is in its infancy so studies which will increase our understanding of this are important for the field. However, this work has several major limitations that limit is potential impact on the field.

We are grateful to this reviewer for these interesting comments and analyses. We have now provided several changes in the manuscript, which included 2 new assays in order to overcome the limitations observed in the first version.

1. It is very descriptive and there is no mechanistic insight into why CTEPH PAEC cells down regulation molecular chaperones in response to flow. This needs to be addressed.

We are grateful for this comment, which helped us looking for EC outcomes relevant to the pathogenesis of CTEPH-EC. It is important to highlight that there is scarce information about CTEPH pathophysiology. Despite thrombus removed with endarterectomy (PEA) residual HP remains in some patients (30%) bring endothelial dysfunction and smooth muscle cell changed to contribute to CTEPH. We have now provided two new experiments to both evaluated endothelial function and HSP70 role through specific HSP70 pharmacological inhibition. The results could be seen below.

**Page 12 / Line 293:** Endothelial dysfunction was proposed to be an essential component in CTEPH (4), however it has not been directly investigated in CTEPH patients. We took advantage of our successful isolation of endothelial cells to evaluate its physiological responses. Interestingly, CTEPH-EC presented strong impairment in migration (Fig. 5) and tube formation (Fig. 6), with less total branches and more cells in each node consistent with frustrated angiogenic response. Moreover, both responses were significantly decreased by the specific (27) HSP70 inhibitor VER155008 (Fig. 5 and 6), clearly showing that HSP70 plays an essential function in migration and angiogenesis both in HPAEC and CTEPH-EC.

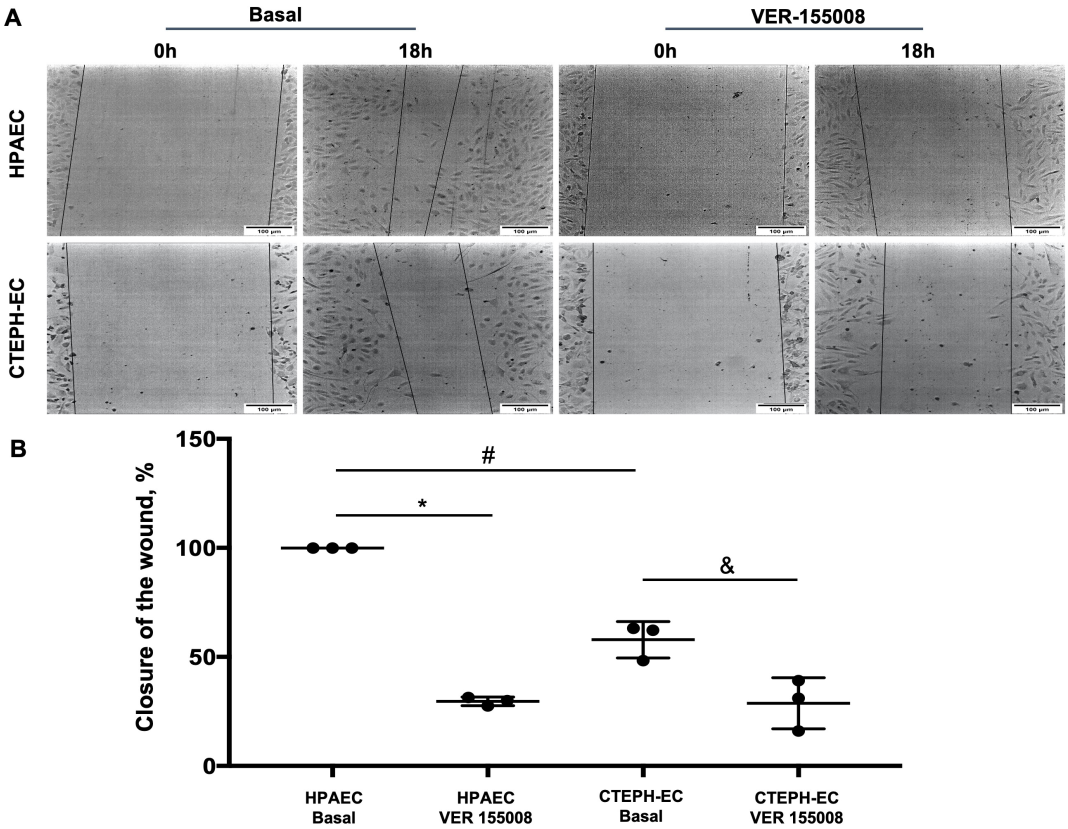


**Fig 5. Migration capacity of HPAEC and CTEPH-EC in basal and with HSP70 inhibitor VER-155008.**

(A) Representative images of HPAECs and CTEPH-ECs basal and after VER-155008. (B) Quantification of the migration. Data are presented as the mean ± standard error: (*) HPAEC Basal vs HPAEC VER, (#) HPAEC Basal vs CTEPH-EC Basal, (&) CTEPH-EC Basal vs CTEPH-EC VER, P < 0.05. (n=3).

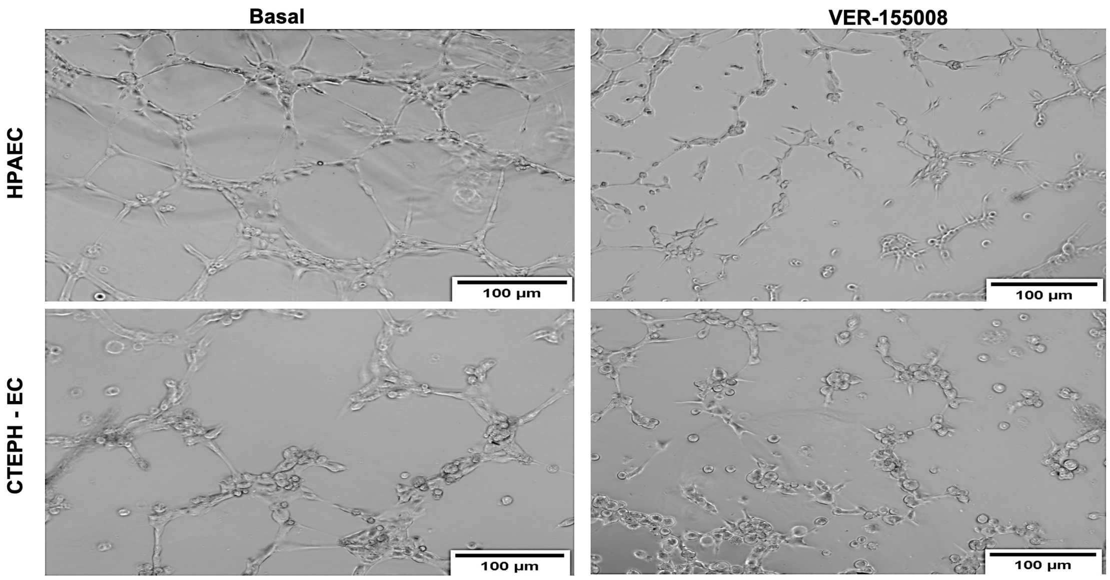


**Fig 6. Tube formation:** representative light microscopic images of the structures formed by HPAEC and CTEPH-EC seeded and incubated for 5h30 min in the absence or presence of 50 uM VER-155008 (n=6).

2. There are actually no studies presented that actually study proteostasis in the manuscript. It would seem that the authors need to evaluate at least a few targets of hsp70/hsp90 or other chaperones and see how these differ in EC isolated from CTEPH patients compared to normal control PAEC.

We have now modified the manuscript and significantly abolished the discussion about proteostasis since we did not, in fact, focus on this theme. The suggestion to evaluate a few HSP70/HSP90 targets in interesting. However, we hope this reviewer will understand that we intend to do this in the future when surgery schedule reopens at our Institution since it stopped after COVID-19 pandemic.

We showed below that eNOS is decreased in CTEPH-EC, but additional assays are necessary to evaluate its functional implications, including phosphorylation and NO production.

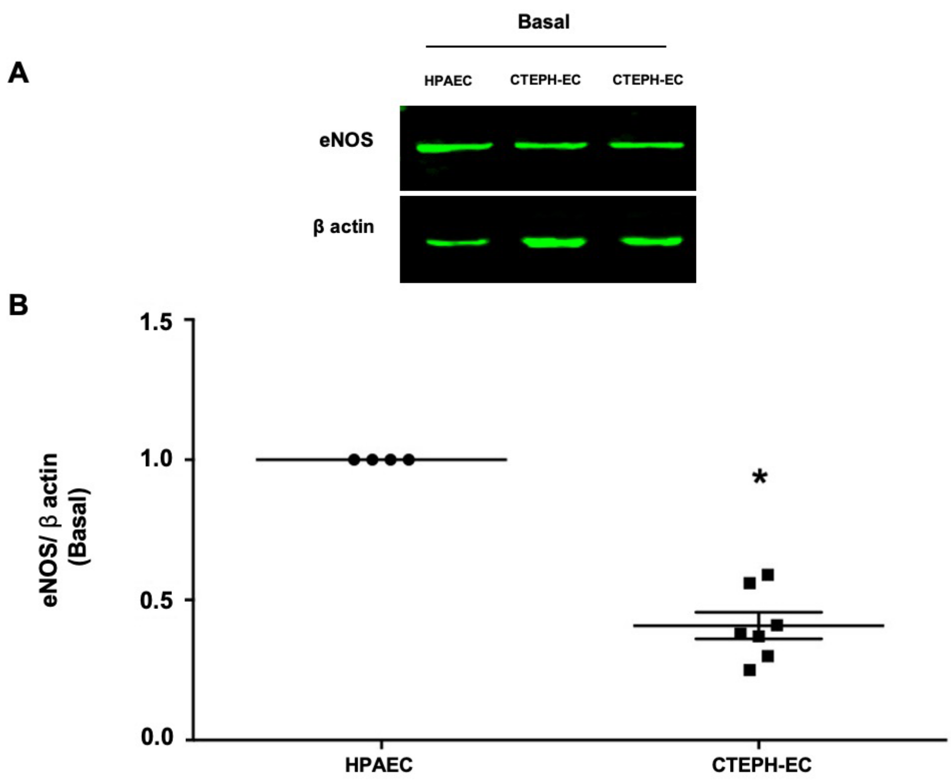


**eNOS expression**

(A) Representative blot of eNOS expression in basal condition

(B) (*) HAPEC vs CTEPH-EC, unpaired Student t test, p<0.05.

3. Can over or under expression of hsp70/hsp90 or other chaperones be used to restore proteostasis in EC from CTEPH patients or perturb it in normal control PAEC respectively? This is needed to demonstrate cause and effect relationships.

We are grateful for this interesting suggestion. Indeed, we have now performed new experiments with HSP70 inhibition, as shown below, and showed that HSP70 is a master regulator of cell function in both HPAEC and CTEPH-EC. On the other hand, HSP70/HSP90 overexpression is not technically feasible, since we have limited quantity of CTEPH-EC and -again- there is no institutional deadline to back to the surgery routine to allow a large number of experiments necessary to try overcoming these limitations.
